# Thermal ablation for pulmonary oligometastases from hepatocellular carcinoma: initial experience and retrospective study

**DOI:** 10.1186/s40644-025-00896-8

**Published:** 2025-06-18

**Authors:** Rongna Hou, Xueliang Zhou, Yipu Li, Yamin Qin, Mengyao Song, Chengzhi Zhang, Zhanguo Sun, Dechao Jiao

**Affiliations:** Department of Interventional Radiology, The First Affiliated Hospital of Zhengzhou University, 1 Jianshe East Road, Zhengzhou, 450052 China

**Keywords:** Thermal ablation, Hepatocellular carcinoma, Pulmonary oligometastases, Overall survival, Progression-free survival

## Abstract

**Objective:**

To evaluate the long-term efficacy of thermal ablation in the treatment of pulmonary oligometastases (POs) from hepatocellular carcinoma (HCC) and to explore the prognosis-related influencing factors.

**Methods:**

From October 2012 to January 2019, 145 POs (mean diameter: 2.3 cm, ≤ 4 POs per patient) in 62 patients (male = 33, female = 29, mean age: 61.0 years old) with HCC were treated by thermal ablation. The primary endpoints were progression-free survival (PFS) and overall survival (OS), and the secondary endpoints were technical success, technical efficacy, and complications. PFS and OS were analyzed by the log-rank test and Cox proportional hazards regression models.

**Results:**

Technical success, technical efficacy and major complications were 100, 96.8, and 21%, respectively. During the median follow-up of 30 months (range: 16–50), the median PFS was 11.4 months (95% CI 10.1–12.8), the 1- and 2-year PFS rates were 43.5 and 10.2%, respectively, and radical treatments for primary HCC (*P* < 0.01), metachronous POs (*P* < 0.01) and initial Barcelona Clinic Liver Cancer (BCLC) stage 0-B (*P* < 0.05) were significant indicators of superior PFS. The mOS was 33.0 months (95% CI 26.9–39.1), and the 1-, 2- and 3-year OS rates were 98.4, 78.7% and 43.7%, respectively. Radical treatments for primary HCC (*P* < 0.01) and initial BCLC stage 0-B (*P* < 0.05) showed superior OS.

**Conclusion:**

POs ablation after primary HCC control is safe and effective, and initial BCLC stage evaluation and radical treatment strategies should be emphasized. This study has certain limitations, including the retrospective design, single-center data, selection bias and small sample size.

## Introduction

Hepatocellular carcinoma (HCC) remains a leading cause of cancer-related mortality globally, with pulmonary metastases occurring in over 50% of advanced cases [1, 2]. Considering that 80% of patients are in an advanced stage when diagnosed, oncologists mainly focus on the local control of primary HCC but pay less attention to lung metastasis or even ignore it. Primary HCC has benefited from radical treatments such as liver transplantation [3], hepatectomy [4], thermal ablation [radiofrequency (RFA) or microwave ablation (MWA)] [5] and palliative treatments such as transarterial chemoembolization (TACE) [6], hepatic artery infusion chemotherapy (HAIC) [7], and targeted drugs [8] (e.g., sorafenib, lenvartinib, and apatinib), which has made significant progress in different Barcelona Clinic Liver Cancer (BCLC) stages of HCC. Survival prolongation is accompanied by a more frequent incidence of lung metastasis; thus, the resolution of lung metastasis has become an increasingly important challenge.

Oligometastases, which were first expressed by Hellman and Weichsel Baum in 1995 [9], refer to an intermediate state between local primary tumors and extensive metastatic tumors (no more than 2 metastatic organs and 5 metastatic lesions). At the early stage of tumor metastasis, because of the weak invasiveness and metastatic capacity of the tumor, metastases are limited to individual organs with limited metastases. The metastases are characterized as two subtypes: synchronous and metachronous metastases; the former refers to metastases occurring synchronously with the primary tumor diagnosis, and the latter refers to metastases occurring after the primary tumor is controlled. The concept of oligometastases represents a change in the traditional model, which previously posited that metastases indicate a terminal advanced stage without the possibility of a radical cure, so palliative systemic treatments have become one of the few options. The effect of local therapies such as surgery, stereotactic body radiotherapy (SBRT) and ablation has been greatly enhanced in patients with pulmonary oligometastases (POs) in recent years. Some studies have confirmed that pneumonectomy can yield survival benefits, but the associated high requirements(such as tumor staging, general condition, anesthesia risk, etc.) and low cost-effectiveness for patients limit its wide clinical application [10]. SBRT for lung metastasis has accumulated much clinical data and has gradually been accepted by most patients. A recent meta-analysis from Lehrer EJ [11] showed that 1-year local control, progression-free survival (PFS) and overall survival (OS) were 67.5–100%, 33.3–80.0% and 65.9–100%, respectively, in 21 studies based on 15 years comprising 943 patients and 1290 POs.

Thermal ablation, such as MWA or RFA, has been a hot research topic abroad in the past 30 years. It is primarily performed by inserting thin puncture needles into tumors, and external energy is applied to tumor tissues through transmission lines. The increased temperature in the region (coagulation necrosis can occur immediately at 60°) will result in irreversible coagulation necrosis of tissues [12, 13]. Compared with SBRT, thermal ablation has the advantages of a short treatment cycle, low cost, and easy implementation, as SBRT is expensive and only used in regional medical centers in China [14]. Besides, according to National Comprehensive Cancer Network (NCCN) Guidelines, for patients with pulmonary metastases who have been carefully selected, if the technique is feasible and is expected to provide long-term local control, ablation therapy (such as radiofrequency ablation, microwave ablation, or cryoablation) can be an alternative treatment option beyond surgery, but few studies have focused on the long-term efficacy of ablation for POs from HCC [15]. A systematic review [16] on microwave ablation for the treatment of colorectal pulmonary metastases indicates that survival post ablation at 1 and 3 year was 89.2% and 40.3%, and post-ablation disease-free survival was 43.2% at 3 years. Thus, this retrospective study aimed to evaluate the long-term results of this treatment and to explore the prognosis-related influencing factors.

## Materials and methods

### Study subjects

**Fig. 1:**
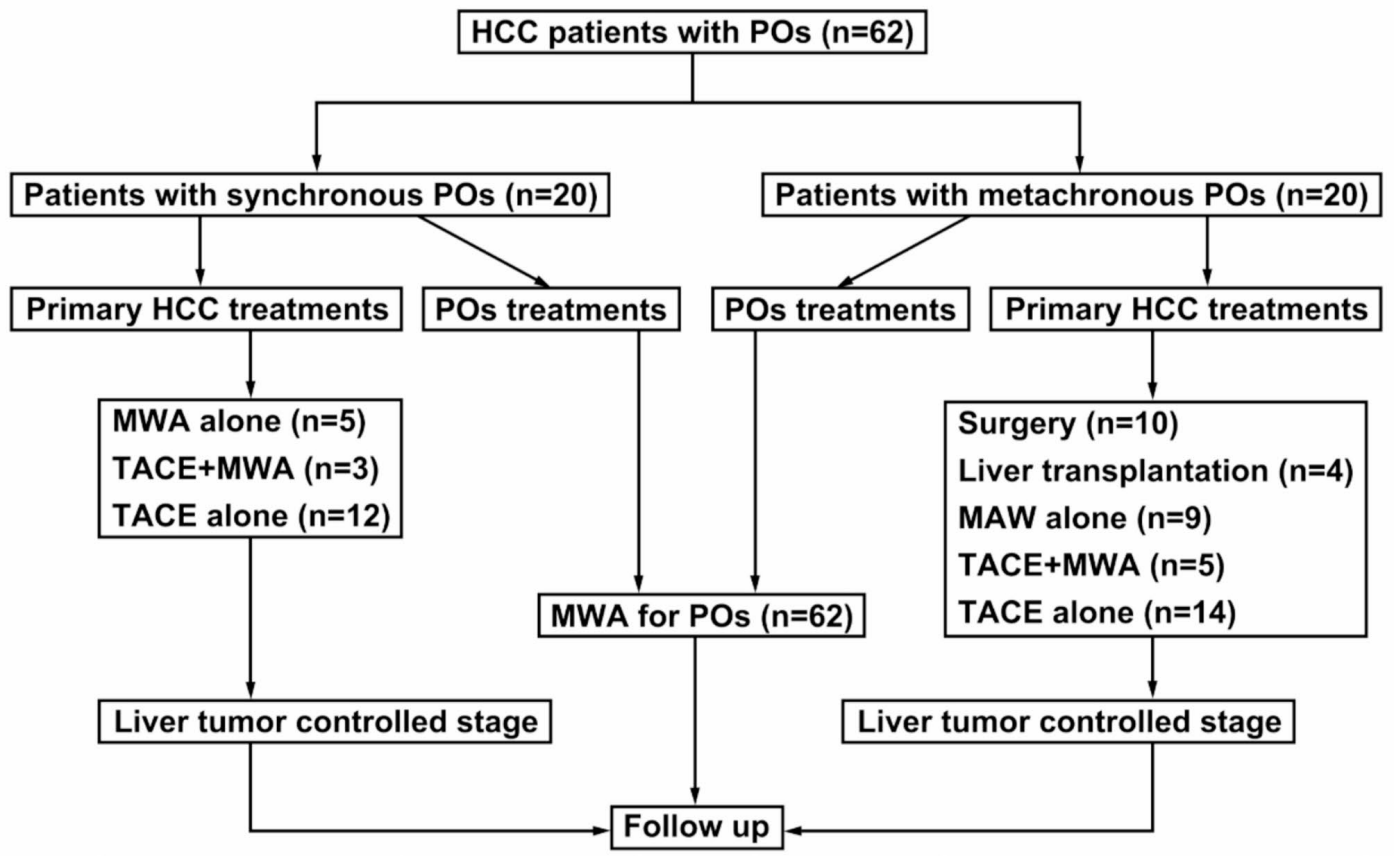
The workflow diagram of this study

In this retrospective study, clinical data from 2236 HCC patients in our center were collected and analyzed between January 2016 and January 2021. The final 62 HCC patients with POs [mean age: (61.0 ± 7.7) year old, (range: 42–72); male/female = 33:29] were included in this study according to the corresponding inclusion/exclusion criteria, with a total of 145 POs lesions [(mean diameter: (2.3 ± 0.6) cm, range: 1–4 cm]. The diagnosis of extrahepatic POs in HCC patients depended on the pathology (*n* = 18) or medical image evidence (*n* = 44). Two or more interventional radiologists independently evaluate the imaging data, and any discrepancies are resolved through consensus. The multidisciplinary team (MDT) discusses cases through regular meetings and formulates treatment plans by integrating imaging, pathological and clinical data. The workflow of this study is listed in Fig. 1. The inclusion criteria were as follows: (1) age range 18–80 years old; (2) primary HCC controlled by surgery, ablation, liver transplantation, TACE and combined treatments; (3) only hepatitis B virus-associated HCC was included; (4) max. diameter of the metastases ≤ 4 cm; (5) extrahepatic metastasis involved only lung; (6) total metastases ≤ 5; (7) extrahepatic metastases location suitable for MWA, such as 1 cm from the main bronchus, a major blood vessel or a nerve tract; (8) Child‒Pugh score of 5–7 (score = 7 allowed only in the absence of ascites); (9) initial BCLC staging 0-C (excluding stage C HCC with left or right main portal vein thrombus); and (10) Eastern Cooperative Oncology Group (ECOG) score = 0 or 1. The exclusion criteria were as follows: (1) presence of other malignant tumors except HCC; (2) alcoholic hepatitis or hepatitis C virus-related HCC; (3) insufficient cardiovascular, hepatic and renal function; (4) complicated with severe coagulation dysfunction (PLT count < 30 × 10^9^/L and PT > 25 s); and (5) life expectancy ≤ 6 months. This retrospective study was conducted in accordance with the Declaration of Helsinki (as revised in 2013). The study was approved by the Ethics Committee of the First Affiliated Hospital of Zhengzhou University (No. 2021-ky-016). Individual consent for this retrospective analysis was waived.

### MWA or RFA procedures

**Fig. 2:**
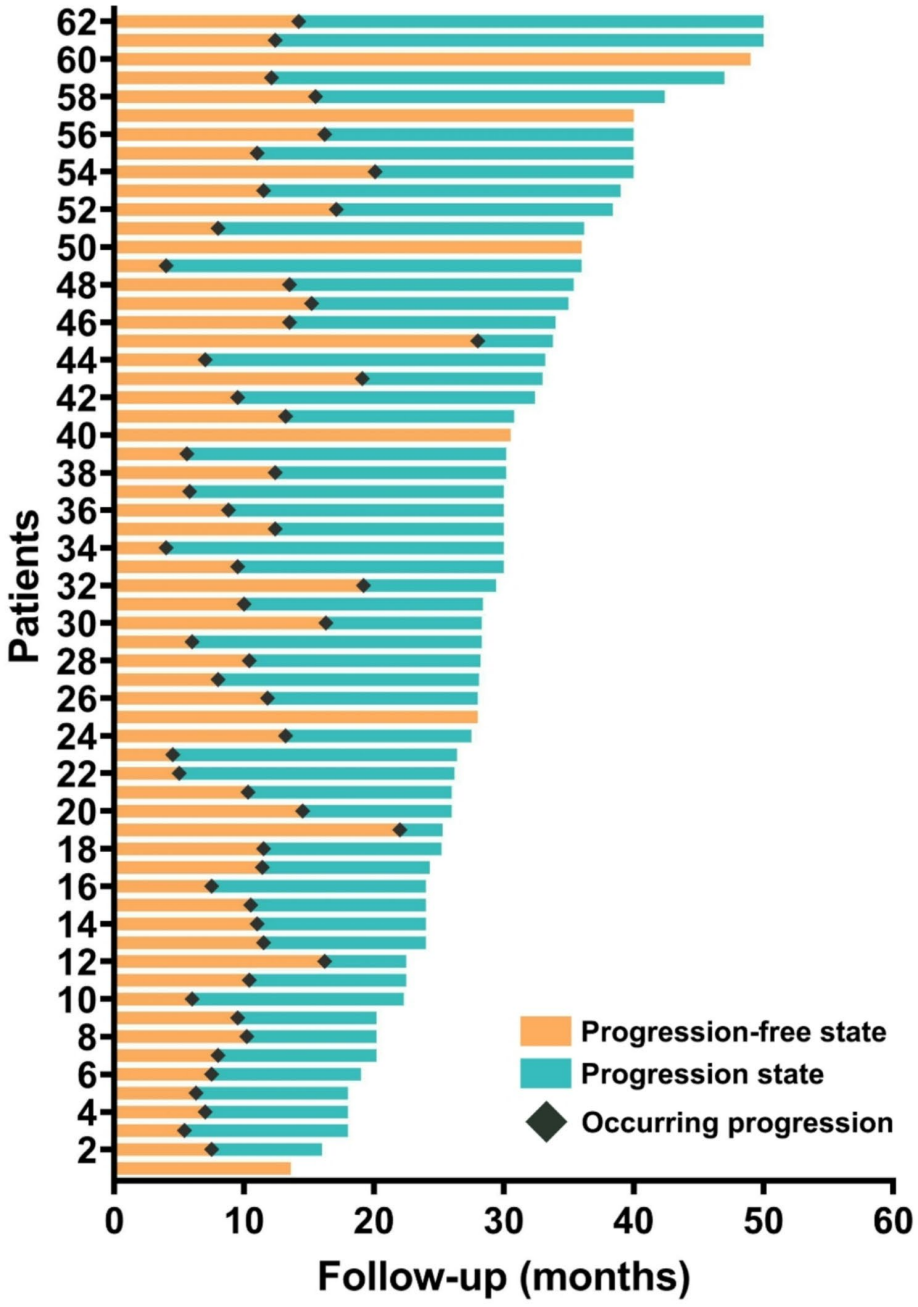
The progression free and disease progression state of all 62 patients

Routine blood tests, liver/kidney function, electrolytes, coagulation function, serum tumor markers such as alpha-fetoprotein (AFP), electrocardiography and abdominal/lung enhanced CT were performed 3–5 days before the operation. Dexmedetomidine (0.5 µg/kg) and dezocine (10 mg) were used intravenously to achieve a satisfactory sedation state, and general anesthesia was used if the patients could not subjectively or objectively tolerate pain. All MWA/RFA procedures were performed under CT guidance (Philips Brilliance 16, The Netherlands; current: 90 mA; voltage: 120 kV and thickness: 3 mm) by two interventional radiologists (Jiao D and Du K, who had 15 and 10 years of experience in puncture, respectively). The typical MWA/RFA procedures included tumor localization, puncture, ablation, and evaluation, as described in our previous study [17, 18]. The design of the puncture approach is based on the principle of puncture convenience and avoidance of important structures, such as blood vessels, nerves, and the main trachea. The MWA generator (Eco Co., Ltd, Nanjing, China) with an adjustable power range of 0–100 W has its own matching MWA applicator [size was (1.8 or 2.0) mm in diameter and (150 or 200) mm in length] and RFA (Cool-Tip, Medtronic, Inc., USA) equipped with a water-cooling circulation system to reduce skin burns, and ablation parameters were used according to the manufacturer’s recommended protocol and our previous clinical experience. Ablation can be completed once for POs less than 3 cm with appropriate puncture, while for those larger than 3 cm, the “overlapping ablation” mode was superior [19]. The ablation zone was recommended to reach more than 5–10 mm of coverage of the per PO margin on post-ablation CT (Fig. 2). X-ray examination was performed regularly 6–8 h later to evaluate pneumothorax occurrence. If the patient had any signs of exacerbation of dyspnea, CT or X-ray examination was performed, followed by immediate treatment.

### Definition and follow-up

Radical treatments included surgery, ablation, and liver transplantation (LT), and palliative treatments included TACE and other combined treatments. Technical success was defined as the successful completion of the ablation operation. Technical efficacy referred to complete ablation of the POs during the follow-up period. Complete ablation (CA) was defined as a typical enhanced CT presentation, such as fibrotic progression, solid nodule involution, cavitation, disappearance, and atelectasis. Incomplete ablation was defined as remaining solid residues, partial fibrosis with solid residues, and increased size with irregular peripheral enhancement on subsequent CT according to the consensus standard of experts in the ablation field [20]. The boundary between high and low hepatic B virus replication was 1 × 10^5^ copies/ml. Targeted treatments refer to treatment with sorafenib or levantinib. Progressive disease (PD) was defined as any tumor recurrence or metastasis after CA of the POs. PFS was defined as the time from the date of entry into the study to the first documented date of PD or death or to the end of the study. OS was defined as the time from the date of entry into the study to the date of death or the last follow-up visit. Major complications were those that resulted in prolonged hospitalization or dysfunction according to the Cardiovascular and Interventional Radiological Society of Europe (CIRSE) classification of Grade 4–6. Minor complications were those with a CIRSE classification of Grade 1–3 [21]. The follow-up period was defined as the time from the date of the ablation procedure until the last visit or death. All patients were followed up every 1 month during the first three months and then every 2–3 months thereafter. The contents of follow-up included routine blood tests, liver and kidney function, AFP, and abdominal/lung enhanced CT.

### Statistical analysis

SPSS 21.0 software (SPSS, Inc., Chicago, IL) was used to perform all statistical analyses. All continuous data are expressed as the mean ± standard deviation (SD). PFS and OS were calculated and compared by the Kaplan‒Meier method and log-rank test. The univariate Cox proportional hazards regression model was used to analyze the factors influencing PFS and OS, subsequently incorporating significant difference factors into the multivariate Cox proportional hazards regression model to identify independent prognostic factors. Hazard ratios with 95% confidence intervals were calculated by a backward elimination approach, and *P* < 0.05 was considered statistically significant.

## Results

### Study population

Of all 62 patients, the primary HCC [BCLC stage 0-B/C was 33 (53.2%) and 29 (46.8%) patients, respectively] was controlled as CA confirmed by enhanced CT or MR by TACE alone (*n* = 26), MWA alone (*n* = 14), TACE + MWA (*n* = 8), surgery (*n* = 10), and liver transplantation (*n* = 4). Among all 29 patients with initial BCLC stage C, 20 patients had synchronous POs, and 9 patients had tumor thrombi of the small branch of the portal vein, which could be ablated by MWA or TACE combined with TACE. There were 1, 2, 3, and 4 POs in 15 (24.2%), 12 (19.4%), 34 (54.8%) and 1 (1.6%) patients, respectively. The subtypes of synchronous and metachronous POs were found in 20 (32.3%) and 42 (67.7%) patients, respectively. 43 (69.4%), 18 (29.0%) and 1 (1.6%) patients were classified as Child‒Pugh class 5, 6, and 7, respectively. Pretreatment high and low virus replications were found in 17 (27.4%) and 45 (72.6%) patients, respectively. Liver cirrhosis status was confirmed by MR/CT findings (*n* = 36) or pathology after surgery (*n* = 14) and percutaneous biopsy (*n* = 12). There were 24 patients (38.7%) who accepted targeted treatment (7 and 17 patients using sorafenib and lenvartinib, respectively) during the follow-up (detailed information is presented in Table 1).

**Table 1:** Baseline characteristics of the patients

Characteristics	Value (%)
Total patients/PO number	62/145
Number of patients with PO (1/2/3/4 metastases)	15(24.2%)/12(19.4%)/34(38.8%)/1(1.6%)
Sex(male/female)	33(53.2%)/29 (46.8%)
Age (mean ± SD, years old)	61.0 ± 7.7
Age stratification (≤ 60/>60 years old)	18(29.0%) / 44(71.0%)
Hepatitis B virus status (≤ 1 × 10^5^/>1 × 10^5^ copies/ml)	45(72.6%)/17(27.4%)
AFP level (≤ 200/>200 ng/ml)	24(38.7%)/38(61.3%)
Liver cirrhosis (yes/no)	47(75.8%)/15(24.2%)
Child-Pugh class (5/6/7)	43(69.4%)/18(29.0%)/1(1.6%)
Initial BCLC stage at diagnosis (Stage 0-B/C)	33(53.2%)/29 (46.8%)
ECOG score (0/1)	31(50.0%)/31(50.0%)
POs sub-type (synchronous/metachronous)	20 (32.3%)/42(67.7%)
Max. diameter of POs stratification (mean ± SD, cm)	2.3 ± 0.6
Max. diameter stratification (≤ 2.5/>2.5 cm)	34(54.8%)/28(45.2%)
Number of POs treated (≤ 2/>2 nodules)	27(43.5%)/35(56.5%)
Primary HCC treatments (radical/palliative treatments)	36(58.1%)/26(41.9%)
Ablation tool for PO (MWA/RFA)	31(50.0%)/31(50.0%)
Target treatments (yes/no)	24(38.7%)/38(61.3%)
Time to disease progression (≤ 12/>12 months)	35(56.5%)/27(43.5%)
PFS (months) (median, 95%CI)	11.4 (95%CI:10.1–12.8)
OS (months) (median, 95%CI)	33.0 (95%C:26.9–39.1)

PO: Pulmonary oligometastases; ECOG: Eastern Cooperative Oncology Group; PFS: progression free survival; OS: overall survival

### Technical efficacy and time to progression

**Fig. 3:**
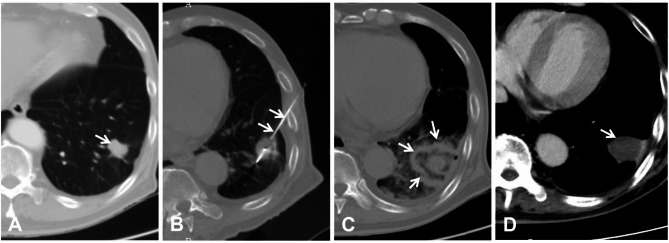
A 65-year-old male with left solitary PO from HCC, AFP: 98 ng/ml. (**A**) Pre-treatment CT showed left solitary PO with **a** diameter of 2.2 cm (arrow); (**B**) microwave applicator (arrow) was inserted into the PO; (**C**) The PO and its around 1.0 cm was completely ablated (40 W/3min) on CT (arrow); (**D**) Post-treatment CT at 4-month showed solid nodule involution without enhancement (arrow)

During the median follow-up of 30 months (range: 16–50), the median PFS was 11.4 months (95% CI 10.1–12.8), and the mOS was 33.0 months (95% CI: 26.9–39.1). In principle, 2 POs should be treated in batches to avoid late fatal bilateral pneumothorax. Technical success was achieved in 106 procedures performed to treat 145 POs, corresponding to a technical success rate of 100%. Technical efficacy was achieved in 60 (96.8%) patients, and in the other two patients, another MWA was needed to achieve CA. 56 (90.3%) patients were evaluated for PD involving the liver (*n* = 38), lymph nodes (*n* = 30), lung (*n* = 25), adrenal gland (*n* = 18) and bone (*n* = 15). All further treatments were decided by a multidisciplinary panel composed of liver surgeons, interventional radiologists, infectiologists, pathologists, and radiologists. There were 35 (time to PD ≤ 12 months) and 21 (time to PD > 12 months) patients who experienced disease progression, respectively (Fig. 3). There were 28 (45.2%) patients still alive at the end of the study, and 34 (54.8%) patients experienced death. The reasons were multiple tumor metastasis (*n* = 27), liver failure (*n* = 4), and upper gastrointestinal bleeding (*n* = 3).

### PFS and prognostic factors

**Fig. 4:**
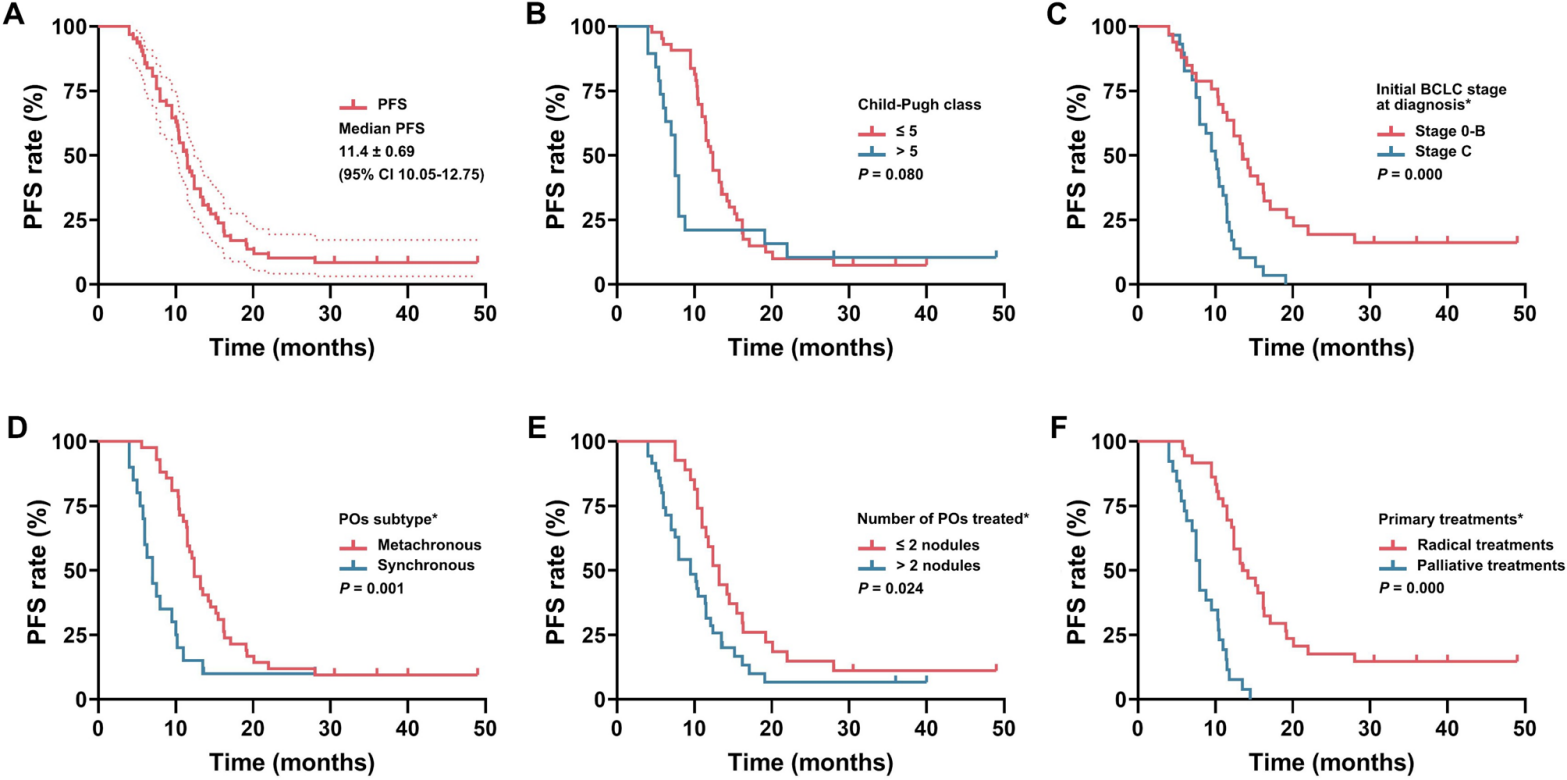
Kaplan-Meier univariate analysis of PFS. (**A**) PFS of all patients; (**B**) Child-Pugh class; (**C**) initial BCLC stage; (**D**) POs subtype; (**E**) number of POs treated per patients; (**F**) treatments for primary HCC. (* indicates significant difference.)

The median PFS was 11.4 months (95% CI: 10.1–12.8), and the 1- and 2-year PFS rates were 43.5% and 10.2%, respectively (Fig. 4). Patients with the following characteristics had superior PFS: metachronous POs [12.40 month (95% CI: 10.92–13.88) vs. 7.00 months (95%CI: 5.48–8.53), *P* = 0.000]; initial BCLC Stage 0-B [13.50 months (95%CI: 11.20–15.80) vs. 10.00 months (95% CI: 8.15–11.85), *P* = 0.000]; 1–2 POs [13.20 months (95% CI: 11.43–14.97) vs. 9.50 months (95%CI: 7.18–11.82), *P* = 0.021]; and radical treatments for primary HCC [13.50 months (95%CI: 10.28–16.72) vs. 8.0 months (95%CI: 7.18–8.82), *P* = 0.000]. Considering the importance of the Child-Pugh class [Child-Pugh class > 5 vs. ≤5, 7.50 months (95% CI: 6.80–8.20) vs. 12.40 months (95%CI: 11.25–13.55), *P* = 0.073], the above five factors were included in the multivariate analyses using the Cox hazards model.

Only radical treatments for primary HCC (HR: 7.836, *P* = 0.000), metachronous POs (HR: 4.057, *P* = 0.000) and initial BCLC Stage 0-B (HR: 2.239, *P* = 0.018) were independent factors for superior PFS. The following characteristics were not significantly different in the univariate analysis: sex (*P* = 0.192), age (*P* = 0.858), hepatitis B virus replication (*P* = 0.122), liver cirrhosis (*P* = 0.585), ECOG score (*P* = 0.442), max. diameter of POs per patient (*P* = 0.578), AFP level (*P* = 0.273), ablation tool (*P* = 0.326), and targeted therapy (*P* = 0.609) (Tables 2 and 3).

**Table 2:** Univariate Cox-regression analysis of PFS and OS

Factors	PFS (months)		OS (months)		
	Median, (95% CI)	*P*-value	Median, (95% CI)	*P*-value	
Sex		0.192		0.514	
Male	10.40, (8.26–12.54)		32.40, (28.53–36.27)		
Female	12.40, (10.83–13.98)		33.80, (29.97–37.54)		
Age		0.858		0.119	
≤ 60 years old	10.40, (10.32–12.48)		33.78, (29.93–37.64)		
> 60 years old	11.40, (6.45–14.35)		39.66, (33.51–45.82)		
Hepatic B virus status		0.122		**0.000**	
≤ 1 × 10^5^ copies/ml	11.50, (10.56–12.44)		28.30, (26.59–30.01)		
> 1 × 10^5^ copies/ml	8.00, (7.34–8.67)		36.00, (28.01–43.99)		
AFP level		0.273		0.872	
≤ 200 ng/ml	11.50, (10.16–12.84)		32.40, (26.55–38.25)		
> 200/ng/ml	10.50, (8.24–12.77)		33.00, (26.21–39.79)		
Liver cirrhosis		0.585		0.746	
Yes	11.00, (6.33–15.67)		33.80, (27.96–39.64)		
No	11.50, (10.16–12.84)		33.00, (27.64–38.36)		
Child-Pugh class		0.073		**0.029**	
Score = 5	12.40, (11.25–13.55)		27.03, (23.12–30.94)		
Score > 5	7.50, (6.80–8.20)		40.41, (36.42–44.41)		
Initial BCLC stage		**0.000**		**0.002**	
Stage 0-B	13.50, (11.20–15.80)		40.00, (30.66–49.34)		
Stage C	10.00, (8.15–11.85)		28.30, (26.91–26.70)		
ECOG score (0/1)		0.442		0.970	
Score 0	12.1, (9.05–15.15)		30.80, (25.71–35.89)		
Score 1	11.00, (8.94–13.06)		33.00, (25.23–40.77)		
POs sub-type		**0.000**		**0.007**	
Synchronous	7.00, (5.48–8.53)		28.30, (24.27–32.33)		
Metachronous	12.40, (10.92–13.88)		36.20, (27.30–45.10)		
Max. Diameter of per patient		0.578		0.337	
≤ 2.5 cm	10.40, (8.12–12.68)		30.00, (27.77–32.23)		
> 2.5 cm	11.50, (10.93–12.07)		36.00, (31.24–40.76)		
Number of POs per patient		**0.021**		0.147	
≤ 2 POs	13.20, (11.43–14.97)		33.80, (20.53–47.07)		
> 2 POs	9.50, (7.18–11.82)		30.00, (25.19–34.81)		
Primary HCC treatments		**0.000**		**0.000**	
Radical	13.50, (10.28–16.72)		28.00, (25.42–30.58)		
Palliative	8.0, (7.18–8.82)		49.00, (33.21–64.79)		
Ablation tools for POs		0.326		0.650	
Microwave ablation	11.50, (9.65–13.35)		32.40, (28.62–36.18)		
Radiofrequency	11.00, (9.04–12.96)		36.20, (25.32–47.08)		
Target therapy		0.609		0.803	
Yes	11.00, (9.79–12.21)		32.40, (25.29–39.51)		
No	11.50, (8.62–14.38)		33.80, (28.51–39.09)		

AFP: alpha fetoprotein; BCLC: Barcelona Clinic Liver Cancer; CI: Confidence interval; ECOG: Eastern Cooperative Oncology Group; OS: overall survival; PFS: progression free survival; PO: Pulmonary oligometastases

**Table 3:** Multivariate Cox-regression analysis of PFS and OS

Factors	PFS (months)		OS (months)	
	HR (95% CI)	*P*-value	HR (95% CI)	*P*-value
Hepatic B virus replication (≤ 1 × 10^5^/> 1 × 10^5^ copies/ml)	---	---	0.736 (0.308–1.757)	0.490
Child-Pugh class (= score 5/> score 5)	1.110 (0.543–2.269)	0.774	0.489 (0.203–1.176)	0.110
Initial BCLC stage at diagnosis (Stage 0-B/Stage C)	2.239 (1.145–4.377)	**0.018**	2.465 (1.144–5.309)	**0.021**
POs subtype (synchronous/metachronous)	4.057 (1.987–8.284)	**0.000**	2.012 (0.929–4.385)	0.085
POs number per patient (≤ 2/> 2 POs)	1.384 (0.773–2.480)	0.274	---	---
Primary HCC treatments (radical/palliative treatments)	7.836 (3.418–17.967)	**0.000**	3.872 (1.452–10.327)	**0.007**

BCLC: Barcelona Clinic Liver Cancer; CI: Confidence interval; HR: hazard ratio; OS: overall survival; PFS: progression-free survival; PO: Pulmonary oligometastases

### OS and prognostic factors

**Fig. 5:**
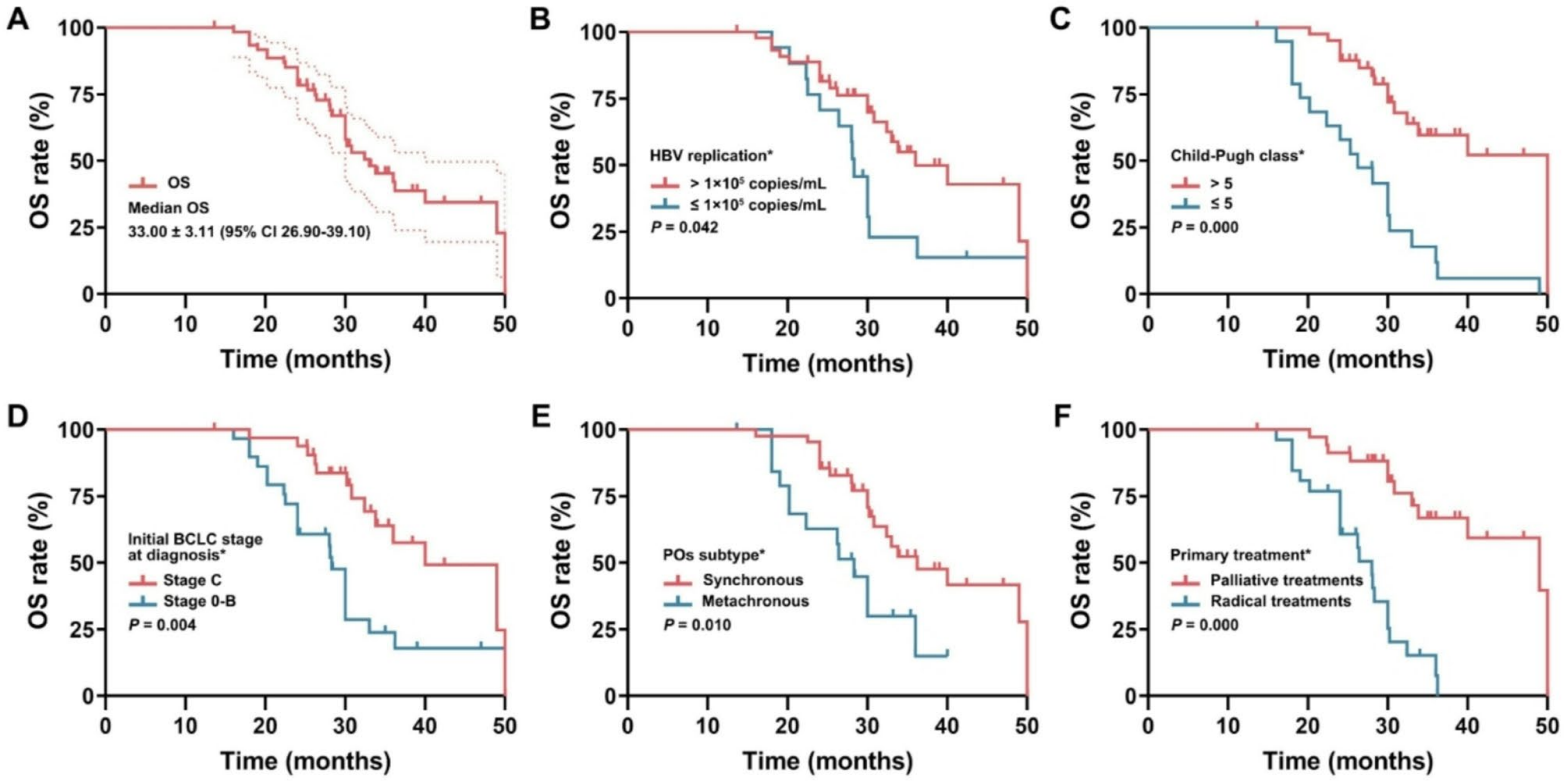
Kaplan-Meier univariate analyses of OS. (**A**) OS of all patients; (**B**) HBV replication; (**C**) Child-Pugh class; (**D**) initial BCLC stage; (**E**) POs subtype; (**F**) treatments for primary HCC. (* indicates significant difference.)

The median OS was 33.0 months (95% CI: 26.9–39.1), and the 1-, 2- and 3-year OS rates were 98.4, 78.7, and 43.7%, respectively (Fig. 5). Patients with the following characteristics had superior OS: Child-Pugh class ≤ 5 [40.57 months (95%CI: 36.61–44.53) vs. 27.92 months (95%CI: 23.55–32.40), *P* = 0.000]; metachronous POs [(36.20 months (95%CI: 27.30–45.10) vs. 28.30 months (95%CI: 24.27–32.33), *P* = 0.026]; low hepatitis B virus replication [36.00 months (95%CI: 28.01–43.99) vs. 28.30 months (95%CI: 26.59–30.01), *P* = 0.028]; radical treatments for primary HCC [49.00 months (95%CI: 33.21–64.79) vs. 28.00 months (95%CI: 25.42–30.58), *P* = 0.000]; and initial BCLC Stage 0-B [40.00 months (95%CI: 30.66–49.34) vs. 28.30 months (95%CI: 26.91–26.70), *P* = 0.002].

The above five factors with a significant difference in the univariate analysis were included in the multivariate analyses. Only primary palliative treatments (HR: 3.872, *P* = 0.021) and initial BCLC Stage C (HR: 2.465, *P* = 0.007) were independent factors for inferior OS. The following characteristics were not significantly different in the univariate analysis: sex (*P* = 0.514), age (*P* = 0.119), liver cirrhosis (*P* = 0.746), ECOG score (*P* = 0.970), number of POs treated (*P* = 0.147), max. diameter of POs (*P* = 0.337), AFP level (*P* = 0.872), ablation tool (*P* = 0.650) and target therapy (*P* = 0.803) (Tables 2 and 3), which might be related to the small sample size or the high homogeneity of the population.

### Complications

The rate of major complications was 21.0%, such as pulmonary infection (*n* = 5), pleural effusion requiring chest tube drainage (*n* = 4), pneumothorax requiring chest tube drainage (*n* = 3), and bronchial fistula (*n* = 1). Minor complications were intrapulmonary hemorrhage (*n* = 38), a small amount of pneumothorax not requiring treatment (*n* = 16), and hemoptysis (≤ 10 ml, *n* = 5). No ablation-associated massive bleeding or death occurred during the peri-ablation period.

## Discussion

Because of the deep anatomical structure and the hidden incidence, 80% of patients with HCC are diagnosed at an advanced stage with portal vein invasion, multiple lesions or multiple organ metastasis, and the prognosis is extremely poor (only 3–6 months without treatment) [22]. A study based on 342 patients by Uchino K [23] showed that the median OS rate was 8.0 months (range: 0.03–108.7 months) in patients with extrahepatic metastases of HCC. The lung is the most common metastatic organ of primary HCC, accounting for 39.5-53.8% of extrahepatic metastases in a previous clinical study [24]. Understanding the mechanism of lung metastasis of HCC will aid in the selection of individualized treatment plans. Currently, the known pathogenesis is presented as follows [25–29]: (1) HCC easily invades the hepatic or portal vein, forming hepatic artery-vein or portal-hepatic vein shunts, such that HCC cells can flow back into the pulmonary circulation through the hepatic vein; (2) the pulmonary circulation is a low-pressure system, with slow blood flow and easy stagnation of cancer cells; (3) the coagulation fibrinolysis activity of lung blood is higher, which is beneficial to the colonization and proliferation of HCC cells; (4) the abnormal expression of genes, proteins or exosomes plays a role in promoting the metastasis and invasion of HCC; and (5) TACE is clinically the most commonly used therapy for HCC at the BCLC A-C Stage, which is associated with the phenomenon of incomplete tumor-feeding arterial embolism, and the residual tumor will stimulate the high expression of vascular endothelial growth factor due to ischemia and hypoxia, thus upregulating the tumor’s invasion and metastasis ability. During clinical practice, because of the shorter OS and higher tumor burden of primary HCC, more clinical studies emphasize the importance of primary site treatment. However, there is no consensus strategy for cases where patients experience lung metastasis at initial diagnosis or after radical treatments such as LT, surgery, ablation, or TACE. Most clinicians believe that HCC enters a state of late systemic blood dissemination, and systemic chemotherapy or targeted therapy are frequently selected, although the benefit (PFS: 1.4–4.2 months) is still low based on a recent meta-analysis [30].

The state of malignant oligometastases refers to the intermediate state between the limited number and spatial distribution of metastatic lesions and progressive lesions (also known as multiple metastases) [31]. Because the biological behavior of tumors is relatively “mild”, local treatments can be more actively selected on the basis of systematic treatments. In the past 30 years, the status of oligometastases or oligoprogression has been confirmed in colorectal cancer, lung cancer, prostate cancer, and other solid tumors [32–34]. Taking liver and lung metastasis of colorectal cancer as an example, local treatments (e.g., surgery, SBRT, and ablation) are not only a model for multidisciplinary comprehensive treatment that is widely accepted by clinicians but have also been included in many international clinical guidelines [35, 36]. Compared with colorectal cancer, HCC is more invasive and has a shorter natural course. Thus, is local ablation for POs meaningful? Even in the latest international guidelines for the diagnosis and treatment of advanced HCC, there is no conceptual definition, diagnostic criteria, differential methods, or treatment specifications.

The present study described the outcome of POs from HCC treated with ablation. The technical success, technical efficacy, and major complication rates were 100%, 96.8%, and 21%, respectively, which were in accordance with the rates in previously published studies (91.0-100%, 61.5–100% and 0-34.3%, respectively) [37]. Compared with a median OS of 8.0 months in Uchino K’s [23] large-sample observational study on extrahepatic metastases from HCC, the median PFS and OS rates of 11.4 and 33.0 months in our study were better. Notably, 9.7% (6/62) of patients survived without disease progression [mean PFS: 32.9 months (range: 13.6–49)], which confirmed that ablation is a potentially curative strategy for selected patients with POs from HCC. Nakamura et al. [38] evaluated pulmonary metastasectomy in 30 patients with HCC. The 1-year, 3-year and median OS rates were 86.7%, 46.2% and 25.0 months, respectively. The study emphasized that use of the appropriate patient selection criteria was the key factor, namely, good general condition, fewer than 3 intrapulmonary metastatic tumors, stable or controllable intrahepatic tumors, limited to one lobe or one lung field, and no extrapulmonary tumors. Jeong YH [39] evaluated the outcomes of pulmonary metastasectomy in 52 patients after LT, and the 1- and 3-year OS rates were 75.0% and 43.5%, respectively, which indicated that a shorter recurrence-free period after LT and adjuvant treatment were independent risk factors. Our study showed that the 1-year, 3-year, and median OS rates were 98.4%, 43.7% and 33.0 months, respectively, which are superior to those obtained in Nakamura [38] and Jeong’s [39] studies. Moreover, the indications for ablation treatment are greater, the complications are fewer, the patient inclusion criteria are fewer, and cost-effectiveness is higher.

The most important finding of the study is that both the initial BCLC stage and treatments independently influence PFS and OS, which means that better PFS and OS can be obtained from initial evaluation and treatment strategies. Although the 29 (BCLC stage C) patients’ primary HCC was mostly controlled by TACE evaluated by enhanced CT, there may be living tumor tissue or HCC stem cells within or at the edge of the embolized zone, so radical treatments such as surgery or ablation are still needed to achieve complete biological necrosis rather than image necrosis. Jansen MC [40] reported that after TACE, a significant tumor response was achieved in 17-61.9% of patients, but a complete tumor response was rare (0-4.8%), as viable tumor cells remained after TACE confirmed by surgery. Frenette CT et al. [41] reported 111 patients with HCC who underwent cTACE (*n* = 76) or DEB-TACE (*n* = 35) before LT, and complete necrosis was achieved in 50.9% and 57.1% of cTACE and DEB-TACE confirmed by pathology, respectively. Chua TC et al. [42] showed that the pathological response of resected specimens was only 27–72% after TACE, although CR was demonstrated on images. Therefore, radical treatments for primary HCC, such as surgery, LT, or ablation, should be performed as soon as possible after TACE, even if CR is evaluated by CT or MR. It is not difficult to understand the impact of POs classification on PFS. Synchronous POs means that HCC cells have been transferred to the lungs through the blood system, and there should be free tumor cells in the circulatory system, which indicates a comparatively higher tumor burden than metachronous POs. That is to say, metachronous POs indicate isolated metastases after the primary lesion has been controlled, and are more suitable for local intervention. Such a phenomenon has been described at POs from lung cancer [43] and colorectal cancer [44] in previous studies. Child-Pugh class was not significant based on multivariate analysis (*P* = 0.11), although it showed significance in the univariate analysis, in contrast to previous studies [45]. The reason may be due to (1) the small sample (*n* = 19) of patients with Child‒Pugh > 5; (2) the improvement of albumin, ascites and other related indicators by conservative treatments; and (3) the fact that the Child‒Pugh class is only a stratification factor based on initial HCC diagnosis, and this indicator changed after surgery (*n* = 10) and LT (*n* = 4) in our study. Overall, this study confirmed that the thermal ablation used for POs is both feasible and safe, consistent with previous reports. It demonstrated that thermal ablation for POs can achieve significantly better survival outcomes than historical data (especially those based solely on supportive treatment or systemic treatment), and it also has the advantage of being minimally invasive.

In conclusion, the study supported the ablation of POs from HCC with good results, and initial BCLC stage evaluation and treatment strategies should be emphasized in our future practice. Given the limitations of this article including a small sample size, retrospective design, single-center data, and selection bias. In the future, multi-center prospective studies need to be conducted to verify the long-term efficacy of thermal ablation in POs and to explore the optimization strategies for combined systemic treatments.

## Data Availability

No datasets were generated or analysed during the current study.
